# A Wide Dynamic Range Sigma-Delta Modulator for EEG Acquisition Using Randomized DWA and Dynamic-Modulated Scaling-Down Techniques

**DOI:** 10.3390/s23010201

**Published:** 2022-12-24

**Authors:** Yongchun Han, Wenhao Liu, Xiangwei Zhang, Xiaosong Wang, Xin Liu, Yu Liu

**Affiliations:** 1 Research and Development Center of Healthcare Electronics, Institute of Microelectronics of Chinese Academy of Sciences, Beijing 100029, China; 2 University of Chinese Academy of Sciences, Beijing 100049, China; 3 Chinese Institute for Brain Research, Beijing 102206, China

**Keywords:** ΣΔ modulator, analog-to-digital (ADC), dynamic range (DR), electroencephalogram (EEG), dynamic-modulated scaling-down (DM-SD), low parasitics capacitance, randomized data weighted averaging (RnDWA)

## Abstract

This paper proposes a wide dynamic range (DR) and high-resolution discrete-time (DT) 2-order 4-bit sigma-delta modulator with a novel dynamic-modulated scaling-down (DM-SD) technology for non-invasive electroencephalogram (EEG) acquisition. The DM-SD technology can expand the input dynamic range and suppress large input offsets at the same time. The modulator was designed with 180nm CMOS technology with an area of 0.49 mm${}^{2}$. We achieve a 118.1 dB SNDR when the input signal is 437.5 Hz and the signal bandwidth is 1500 Hz. Due to the proposed DM-SD technology, the DR is expanded to 126 dB. The power consumption of the whole modulator is 1.6 mW and a 177.8 dB Schreier figure-of-merit (FoMs) is realized.

## 1. Introduction

EEG is a kind of bioelectrical signal corresponding to the brain’s electrical activities. It can reflect whether the physiological activity of the human body is normal. Compared to the traditional diagnostic methods such as X-ray, the non-invasive EEG signal acquisition has the characteristics of no radiation and being non-invasive. As a result, it is widely used in neuroscience research, disease diagnosis, and treatment [1,2,3]. The capture of high-fidelity EEG signals is urgently demanded in brain-controlled applications, such as brain–computer interface (BCI) systems. Since the EEG signal is the weakest bioelectrical signal with extremely low amplitude (1–100 μV), the acquisition system should achieve a high signal-to-noise ratio (SNR) in the analog front end [4,5]. As mentioned in [6], the EEG spikes have a bandwidth of 0.5 Hz–1 kHz, and this requires that the AFE has a kilohertz bandwidth. Moreover, EEG signal is easily affected by undesired interference and noise, such as power line interference, motion artifacts, and offset. So the signal acquisition circuit must have a wide dynamic range (DR) to avoid the circuit entering into saturation [7]. In addition, most EEG signal acquisition systems will use multi-channel acquisition to improve the spatial resolution, low power consumption should also be considered when designing the acquisition circuit.

Many high-performance ADCs for EEG signal acquisition have been proposed to solve the problems mentioned above in the past few years. Among them, the Nyquist-rate successive-approximation-register (SAR) ADC is widely used for EEG signal acquisition due to its high power efficiency and small area. In [8], an SAR ADC using a dynamic random storage unit for biological signal acquisition has been proposed, which achieves 9.26 bits at 26.24uW power consumption. However, the problems caused by capacitance mismatch limit the accuracy of SAR ADC. In the literature [9,10,11,12], various capacitance mismatch cancellation techniques have been used to reduce the impact. However, even with the most advanced calibration techniques, the ENOB of the SAR ADC proposed in these papers is limited to 16 bits due to the limitation of the noise inside the comparator. For higher resolution, the sigma-delta ADC is another option. Due to the use of oversampling technology, the sigma-delta ADC can obtain a higher SNDR. In [13], a 3-order 1-bit continuous-time modulator is proposed. The high-order modulator can cause stability problems and limit the DR of the modulator. Thus, the modulator’s DR only achieves 80 dB. In [14], a 5-bit quantifier continuous-time modulator is proposed to reduce the signal amplitude in the loop and improve the stability of the modulator. However, the continuous-time modulator is susceptible to process, voltage, and temperature (PVT) variation. The proposed modulator achieves a DR of 92.3 dB at 100 Hz bandwidth. In [15], A 3-order DT SDM is proposed. By introducing a resistor, the harmonic is suppressed and a 102.3 dB DR is achieved. However, the structure of the modulator and the setup accuracy of the integrators limit the dynamic range of the modulator. In summary, it is challenging to design an ADC with a wide DR and high resolution while consuming several milliwatts.

To solve the difficulties mentioned above, this paper proposes a DT sigma-delta modulator used for EEG acquisition. We propose the following technologies to improve the performance of the modulator. Firstly, we propose an innovative dynamic-modulated scaling-down (DM-SD) technology to extend the DR of the modulator. A scaling factor of 3/4 is introduced to avoid the modulator entering in saturation or instability state with a full-scale input. Secondly, a low parasitic capacitance integrator by introducing a “negative capacitance” has been proposed to reduce the setting error of the integrators induced by the parasitic capacitance. Thirdly, we propose a simple random circuit and use the randomized data weighted averaging (RnDWA) to reduce the mismatch of the feedback digital-to-analog converter (DAC). Finally, we use the voltage domain technology (VDT) to reduce the power consumption of the quantizer and analysis it in theory. With VDT, the power consumption of the quantizer and logic circuit can reduce by about 30%. The proposed modulator is implemented in 0.18 um CMOS technology with a power supply voltage of 5 V and 1.8 V, occupying an area of 0.49 mm${}^{2}$. The simulation results show that when the input signal is 437.5 Hz with a 1500 Hz signal bandwidth, the proposed modulator can achieve 118.1 dB SNDR. Due to the proposed DM-SD technology, the DR of the modulator has been extended from 120 dB to 126 dB. The total power consumption of the modulator is 1.6 mW, corresponding to 177.8 dB FoMs.

The rest of the paper is organized as follows: In Section 2, the architecture of the proposed modulator is introduced. Section 3 describes the circuit analysis and implementation. The simulation results are shown in Section 4. The conclusion is presented in Section 5.

## 2. System Design

### 2.1. Overview of System Architecture

**Figure 1:**
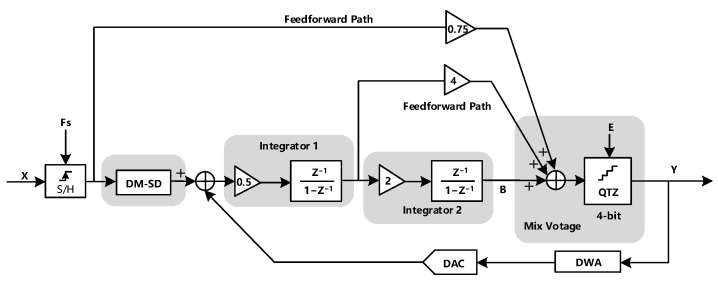
Block diagram of the proposed second-order feed-forward ΣΔ modulator.

The proposed DT ΣΔ modulator is shown in Figure 1. It is a 2-order 4-bit modulator with a 1500 Hz signal bandwidth and is driven by a 1.024 Mhz clock. The modulator consists of the proposed DM-SD module, the low parasitic capacitance integrators, and a 4-bit quantizer. The coefficients of the modulator have been carefully optimized to reduce the output swing of the integrator and save power consumption.

### 2.2. Analysis of Circuit Non-Ideality

The integrator is the crucial part of the modulator, and the noise of the first integrator is important since its noise cannot be shaped by the loop. The noise mainly includes thermal noise, flicker noise, and power noise. The flicker noise can be reduced by chopper technology.

As mentioned in [16,17], the power/ground noise has a spectrum from several kHz to several GHz. The power/ground noise affects any blocks connected to the power distribution network, such as the amplifier and the comparator array. When the frequency of power/ground noise coupled on ADC circuits is mid-band frequency, the ENOB is decreased by about 0.8 bit [16]. We reduce the influence of power noise by using the linear power supply and coupling capacitance.

**Figure 2:**
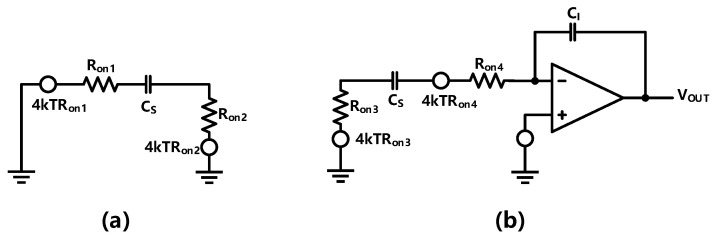
Noise model of the integrator in (**a**) sampling phase (**b**) integrating phase.

The noise model of the integrator is shown in Figure 2,

Figure 2a shows the noise model of the sampling phase. In this phase, the thermal noise of the two switches can be combined, and the power spectral density on the sampling capacitance can be expressed as:

(1) $$S_{C_{S}}\left(f\right)=\frac{8kTR_{on}}{1+{\left(2\pi f\tau \right)}^{2}}$$

where $\tau =2R_{on}C_{S}$ is the time constant, k is the Boltzmann constant of $1.38\times 10^{-23}$ J/K, *T* is the absolute temperature, *f* is the frequency, $C_{S}$ is the sampling capacitance, and $R_{on}$ is the on-resistance of the switch. By integrating the power spectral density over the 3 dB band, and considering the oversampling technology, the noise power in the band is:

(2) $$\bar{v_{C_{S}}^{2}}=\frac{1}{OSR}\int_{0}^{\infty }\frac{8kTR_{on}}{1+{\left(2\pi f\tau \right)}^{2}}df=\frac{1}{OSR}\frac{kT}{C_{S}}$$

The noise model of the integrating phase is shown in Figure 2b. The main noise sources of the integrating phase are the thermal noise from the switches and the amplifier. The amplifier used in the first integrator is a two-stage feedforward miller compensation (FFMC) operational transconductance amplifier (OTA), which can be approximated to a single pole system through careful design. According to [18,19], the noise introduced by the switches can be derived as:

(3) $$\bar{v_{n,C_{S},int}^{2}}=\frac{8kTR_{on}}{4\left(2R_{on}+1/g_{mOTA}\right)C_{S}}=\frac{kT/C_{S}}{1+1/(2g_{mOTA}R_{on})}$$

and the noise introduced by the amplifier is:

(4) $$\begin{array}{c} \bar{v_{n,op}^{2}}=\frac{S_{op}\left(n\right)}{4\tau }=\frac{1}{4\left(2R_{on}+1/g_{mOTA}\right)C_{S}}\left(\frac{8kT\gamma }{g_{mOTA}}\left(1+\frac{g_{m9}}{g_{mOTA}}\right)+\frac{K_{N}}{C_{ox}\left(WL\right)f}\left(1+\frac{g_{m9}^{2}}{g_{mOTA}^{2}}\right)\right)\\ =\frac{8}{3}\frac{kT/C_{S}}{1+2R_{on}g_{mOTA}}\left(1+\frac{g_{m9}}{g_{mOTA}}\right)+\frac{K_{N}g_{mOTA}}{\left(8R_{on}g_{mOTA}+1\right)C_{S}C_{ox}\left(WL\right)f}\left(1+\frac{g_{m9}^{2}}{g_{mOTA}^{2}}\right) \end{array}$$

where γ is coefficient which can be derived to be equal to 2/3 for long-channel transistors, OSR is the oversampling rate, and $K_{N}$ is a process-dependent constant. The total input noise of the integrator can be expressed as:

(5) $$\begin{array}{c} \begin{array}{cccc} \; & \; & \bar{v_{n}^{2}} & =\bar{v_{n,C_{S,samp}}}+\bar{v_{n,op}^{2}}+\bar{v_{n,C_{s},int}}\\ \; & \; & \; & =\frac{kT}{C_{S}}\left(1+\frac{1}{1+1/x}+\frac{3}{3(1+x)}\left(1+\frac{g_{m9}}{g_{mOTA}}\right)\right)+\frac{K_{N}g_{mOTA}}{\left(4x+1\right)C_{S}C_{ox}\left(WL\right)f}\left(1+\frac{g_{m9}^{2}}{g_{mOTA}^{2}}\right) \end{array} \end{array}$$

where $x=2R_{on}g_{mOTA}$, if x≫1, the noise of the integrator is mainly determined by the thermal noise of the sampling capacitor.

We also performed behavior simulations to evaluate the impact of the non-idealities on the SNDR. The non-ideal factors considered here include the finite gain of the OTA, the KT/C noise of sample switches, slew rate (SR), unity-gain-bandwidth (UGB) of the OTA, and mismatch of the DAC capacitors array.

**Figure 3:**
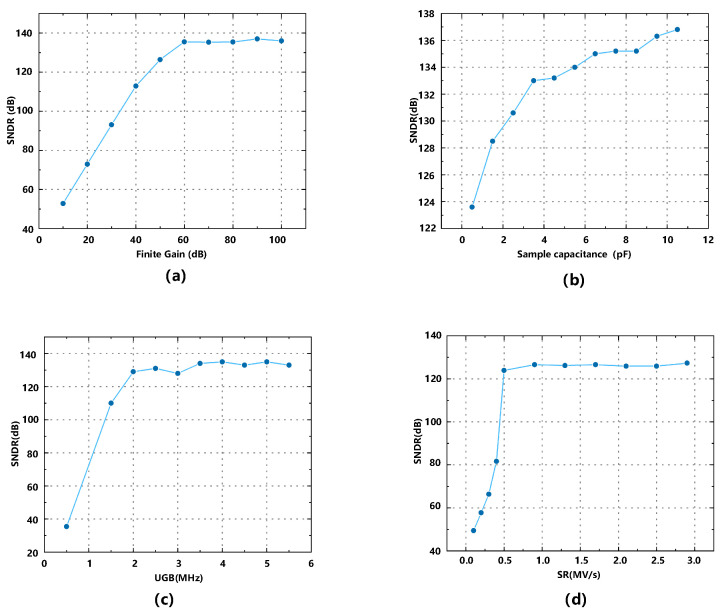
Impacts of circuit non-idealities to SNDR including (**a**) finite gain, (**b**) KT/C noise, (**c**) unit-gain-bandwidth, and (**d**) slew rate.

From the simulation results shown in Figure 3, we can determine the preliminary parameters of the non-ideal factors in the circuit-level design. For example, the finite DC gain of the amplifier should be greater than 60 dB and the sampling capacitance should be greater than 4 pF. Table 1 summarizes the required conditions.

With the conditions in Table 1, the behavior level simulation shows that the structure can achieve 129 dB SNDR at 1500 Hz signal bandwidth.

## 3. Circuit Analysis and Implementation

**Figure 4:**
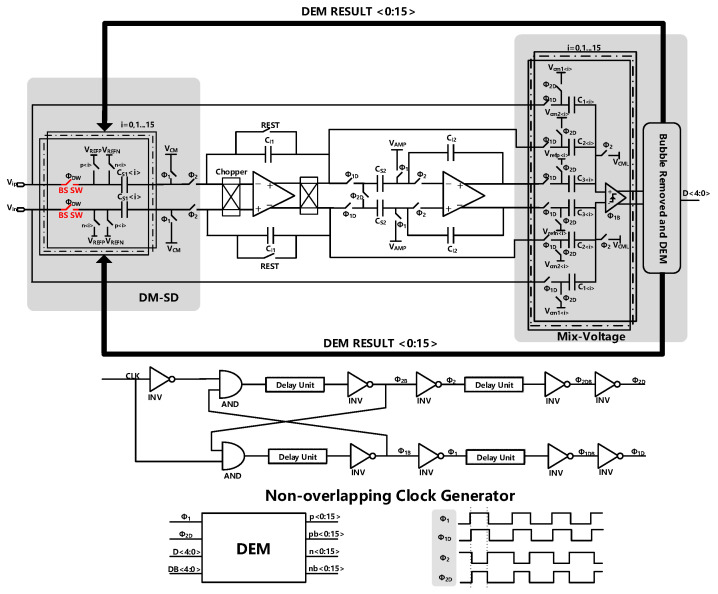
Top-level schematic of the proposed DT ΣΔ modulator and the non-overlapping clock.

The top-level schematic of the proposed modulator is shown in Figure 4. The modulator which is driven by a non-overlapping clock consists of two integrators, the quantizer, and a DAC.

### 3.1. The Proposed Dynamic-Modulated Scaling-Down (DM-SD) Module

**Figure 5:**
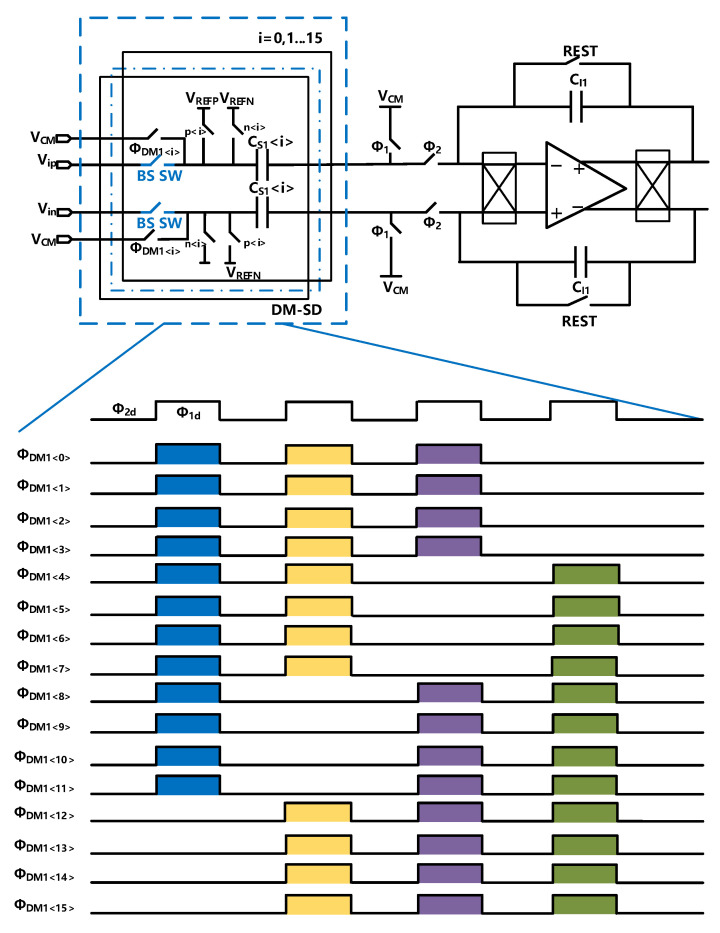
Schematic and timing diagram of the proposed DM-SD.

The schematic and timing diagram of the proposed DM-SD module is shown in Figure 5. A scaling factor of 3/4 is introduced to optimize the signal transfer function (STF) to extend the DR. The scaling factor takes into account the cycle length and the impact on the overall modulator. In this paper, we shared the sampling capacitance with the feedback DAC capacitance array, which is divided into 16 units. If the scaling factor is large, such as 15/16, it takes 16 cycles to complete a cycle, and we need to complete it in the shortest time so that the signal is approximately unchanged. However, if it is too small, the performance of the modulator will be degraded. So, 3/4 is selected as the coefficient. We use 12 capacitors for sampling the input signal in each cycle, and the remaining capacitors are free-wheeling (connected to the common mode voltage $V_{CM}$). A dynamic modulation technology is used to eliminate coefficient mismatch errors. As shown in Figure 5, in cycle one, capacitors $C_{S0}$–$C_{S11}$ are selected and capacitors $C_{S12}$–$C_{S15}$ are free-wheeling. In cycle two, capacitors $C_{S12}$–$C_{S15},C_{S0}$–$C_{S7}$ are selected and capacitors $C_{S8}$–$C_{S11}$ are free-wheeling. In cycle three, capacitors $C_{S8}$–$C_{S15},C_{S0}$–$C_{S3}$ are selected, and in cycle four, capacitors $C_{S4}$–$C_{S15}$ are selected.

The mismatch of the coefficient includes random mismatch and systematic mismatch, assuming that the total element mismatch of the unit is $\delta_{m}=\delta_{Rm}+\delta_{Sm}$, where $\delta_{Rm}$ is the random mismatch and $\delta_{Sm}$ is the systematic mismatching. The capacitance m can be expressed as $C_{m}=C+\delta_{m}$, where C is the ideal capacitance unit. As mentioned above, the average coefficient is equal to equation:

(6) $$\begin{array}{c} \begin{array}{cccc} \; & \; & Coe_{average} & =\frac{1}{4}\left(\frac{\Sigma_{m=0}^{11}C_{m}}{\Sigma_{m=0}^{15}C_{m}}+\frac{\Sigma_{m=12}^{15}C_{m}+\Sigma_{m=0}^{7}C_{m}}{\Sigma_{m=0}^{15}C_{m}}+\frac{\Sigma_{m=8}^{15}C_{m}+\Sigma_{m=0}^{3}C_{m}}{\Sigma_{m=0}^{15}C_{m}}+\frac{\Sigma_{m=4}^{15}C_{m}}{\Sigma_{m=0}^{15}C_{m}}\right)\\ \; & \; & \; & =\frac{1}{4}\frac{(\Sigma_{m=0}^{15}C+\Sigma_{i=0}^{15}\delta_{m})*3}{16C+\Sigma_{m=0}^{15}\delta_{m}}=\frac{3}{4} \end{array} \end{array}$$

From the result, we find that the mismatch of the coefficient can be sufficiently reduced with the proposed DM-SD technology. Furthermore, with the scaling factor of 3/4 introduced by the proposed DM-SD technology, the modulator can withstand full-scale input without being saturated or unstable, leading to extended DR.

### 3.2. Low Parasitic Capacitor Integrator

**Figure 6:**
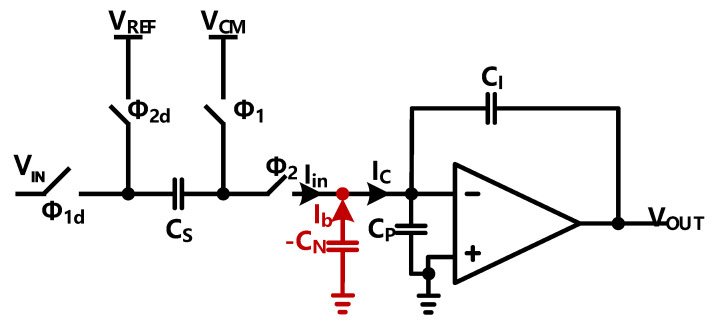
Schematic of the proposed low parasitic capacitance integrator.

The proposed low parasitic capacitor integrator in this paper is shown in Figure 6. For simplicity, we hereby draw the single-ended version instead of the fully differential one in the real implementation.

**Figure 7:**
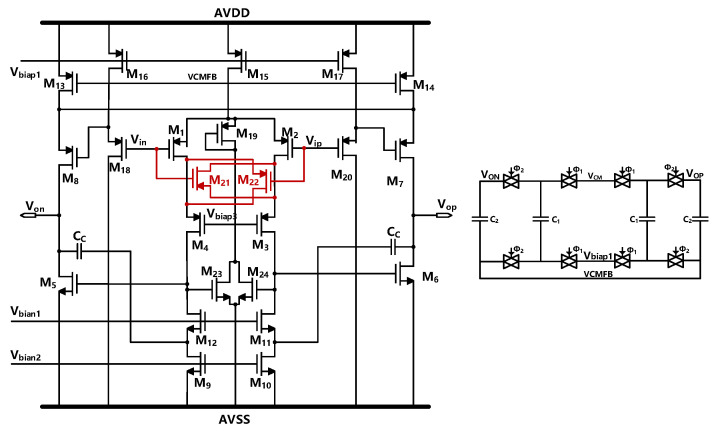
Schematic of the FFMC OTA used in the first integrator.

**Figure 8:**
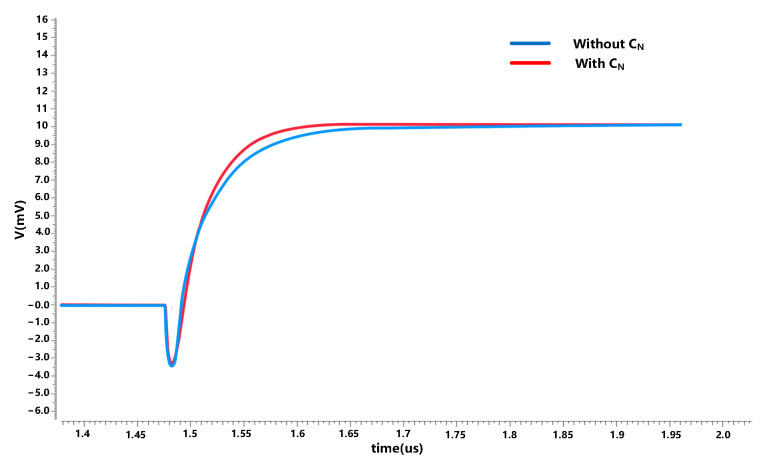
The output voltage with/without $C_{N}$.

As mentioned in [20,21], the output of the integrator during integration can be derived as:

(7) $$v_{o}\left(t\right)=v_{o}\left(nT_{s}\right)+\frac{C_{S}}{C_{I}}\left(V_{ref}-V_{i}\left[nT_{s}\right]\right)-\left(1+\frac{C_{p}}{C_{I}}\right)v_{a}\left(nT_{s}\right)+\left(1+\frac{C_{p}}{C_{I}}\right)v_{a0}e^{-\frac{g_{m}}{C_{eq,\phi_{2}}}t}$$

where t>nT, $C_{eq,\phi_{2}}=C_{P}+C_{S}+C_{L}\left(1+\frac{C_{P}+C_{S}}{C_{I}}\right)$, and the time constant of this equation is $\tau =\frac{C_{eq,\phi_{2}}}{g_{m}}$, the parasitic capacitance can induce a incomplete setting error. We use a ‘negative’ capacitance which has been merged with the OTA (Figure 7) to reduce the influence of the parasitic capacitance. As shown in Figure 6, the equal parasitic capacitance can be derived. We assume that $V_{in}$ is the voltage of the negative input of the OTA, $I_{C}$ is the current flowing to the $C_{P}$, $I_{b}$ is the current from the $-C_{N}$, and $I_{in}$ is the equivalent current flowing into the negative node of the OTA. So the $C_{eqp}$ can be written as:

(8) $$\begin{array}{c} \begin{array}{cccc} \; & \; & C_{eqp} & =I_{in}/V_{in}\\ \; & \; & I_{in} & =I_{C}-I_{b}=\left(V_{in}C_{P}-\left(0-V_{in}\right)\cdot \left(-C_{N}\right)\right)=V_{in}\left(C_{P}-C_{N}\right) \end{array} \end{array}$$

so that $C_{eqp}=C_{P}-C_{N}$, with the ‘negative’ capacitance the expression can be rewritten as:

(9) $$v_{o}\left(t\right)=v_{o}\left(nT_{s}\right)+\frac{C_{S}}{C_{I}}\left(V_{ref}-V_{i}\left[nT_{s}\right]\right)-\left(1+\frac{C_{p}-C_{N}}{C_{I}}\right)v_{a}\left(nT_{s}\right)+\left(1+\frac{C_{p}-C_{N}}{C_{I}}\right)v_{a0}e^{-\frac{g_{m}}{C_{eq,\phi_{2}}^{\prime }}t}$$

where $C_{eq,\phi_{2}}^{\prime }=C_{P}-C_{N}+C_{S}+C_{L}\left(1+\frac{C_{P}-C_{N}+C_{S}}{C_{I}}\right)$, thus the influence of the parasitic capacitance on setting error can be reduced. Figure 8 shows the transient response with and without ‘negative’ capacitance.

**Figure 9:**
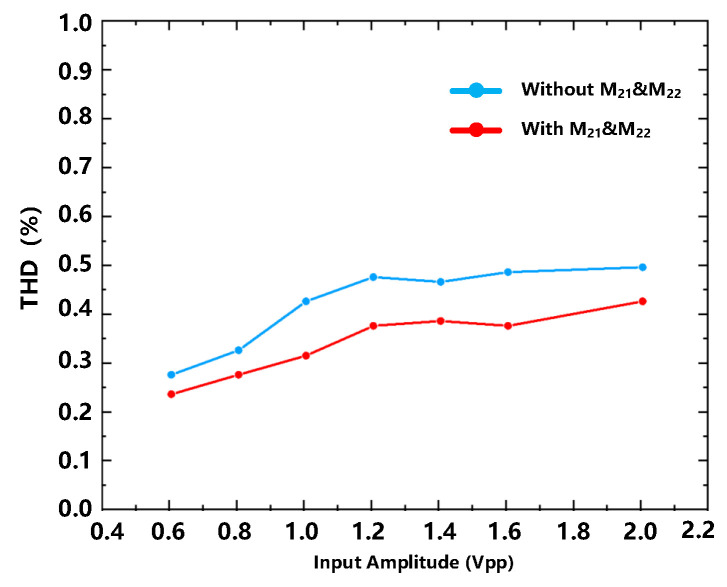
THD (Total Harmonic Distortion)(%) versus input amplitude with/without $C_{N}$.

The result shows that with the ‘negative’ capacitance the output of the integrator can set up faster. Figure 9 summarizes the improvement in linearity due to this effect at different magnitudes.

From Section 2.2, we can conclude that the gain of the amplifier of the first stage integrator should be sufficiently large. We use a cross-coupled FFMC amplifier to obtain higher DC gain [22,23]. The schematic diagram of the amplifier is shown in Figure 7.

**Figure 10:**
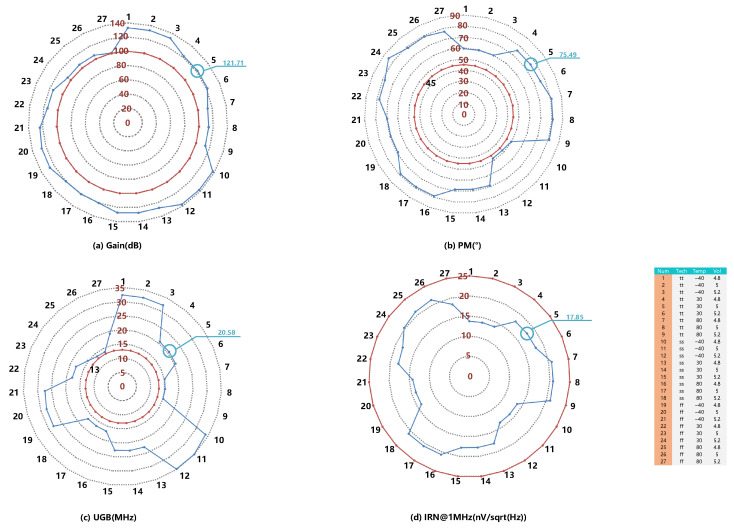
The PVT simulation result of (**a**) gain, (**b**) PM, (**c**) UGB, and (**d**) IRN of the opamp.

To further verify the performance of the first OTA, the specification of gain, PM, IRN, and UGB has been simulated with 27 conditions, including different process corners (tt/ss/ff), temperature (−40/30/80), and voltage (4.8/5/5.2 V). the simulation result is shown in Figure 10. With a 5pF load capacitance, the OTA achieved a 121.71 dB DC gain, 75.49 dB phase-margin, and 20.65 MHz unity-gain-bandwidth with 17.85 nV $\sqrt{Hz}@1MHz$ input-referenced noise. The simulation result shows that the OTA can meet the requirements listed in Table 1 even in the worst case.

**Figure 11:**
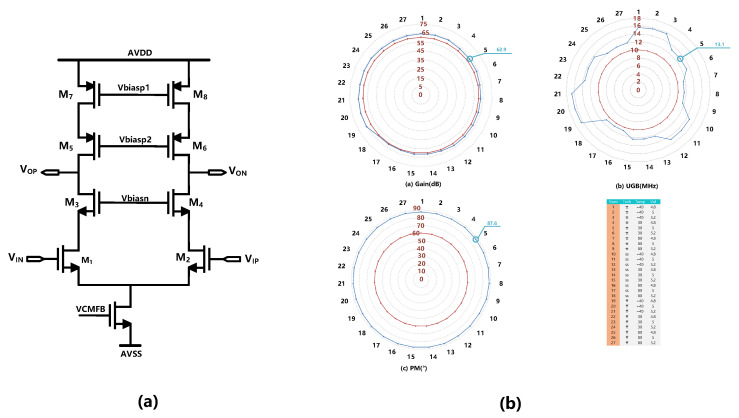
(**a**) Schematic of the second OTA. (**b**) The PVT simulation result of the second OTA.

Since the non-ideal factor of the second stage integrator can be shaped by the loop, the requirement of the second OTA can be significantly relaxed. The telescopic OTA is used to save power consumption. The schematic diagram and simulation results of the OTA are shown in Figure 11. The simulation results show that the gain of the second stage amplifier is greater than 60 dB and the gain bandwidth product is greater than 10 MHz.

### 3.3. The Randomized Data Weighted Averaging (RnDWA)

**Figure 12:**
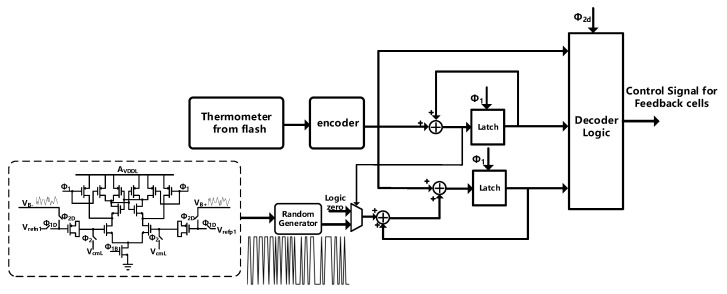
The structure of RnDWA module.

The use of a multi-bit quantizer will introduce the problem of capacitance mismatch. We use RnDWA technology [24,25] to reduce the influence of capacitance mismatch. The structure diagram of the RnDWA is shown in Figure 12. A simple random number generator circuit is presented, which makes use of the characteristics of the proposed modulator to generate random signals. As shown in Figure 1, the voltage $V_{B}$, which is the output voltage of the second integrator, can be expressed as $E\left(Z\right)Z^{-2}$ and it is a delay version of the quantization noise which is random noise. The voltage $V_{B}$ is compared with a reference voltage using a comparator to convert the noise to digital code. Since the accuracy requirement of the comparator is not high, the comparator can be designed with minimum size to save area. Compared with traditional random generation circuits [26,27,28,29], the complexity and the occupied area are reduced. The total additional area for the random generator (including the comparator) is 20 μm${}^{2}$. Compared with the traditional random generation circuits, the area has been reduce at least 60%.

**Figure 13:**
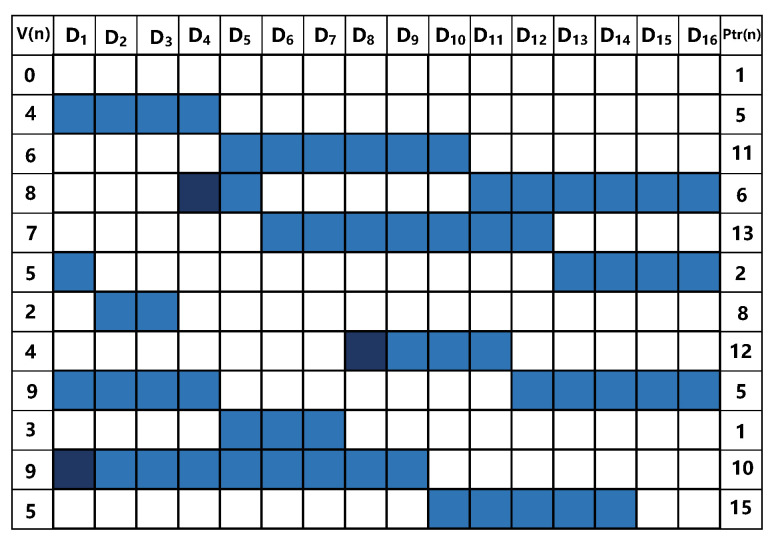
Demonstration of the RnDWA algorithm, the left-most (V(n)) column is the input data sequence, and the right-most (Ptr (n) ) column is the position of the pointer.

With the RnDWA, the units of DAC can be selected through the following logic: As with the classical DWA, RnDWA first completes a rotation based on the input sequence and the current pointer. In addition, we then select a random new start point according to the input random number. When this operation is completed, we continue selecting units until we reach the same start point again. Then another new start point is chosen, and so on. For example, we assume that the input sequence is ‘0, 4, 6, 8, 7, 5, 2, 4, 9, 3, 9, 5’, the random number used is ‘3, 4, 9’, and the selected units are shown in Figure 13. The colored square is the currently selected DAC cell and the white is unused.

**Figure 14:**
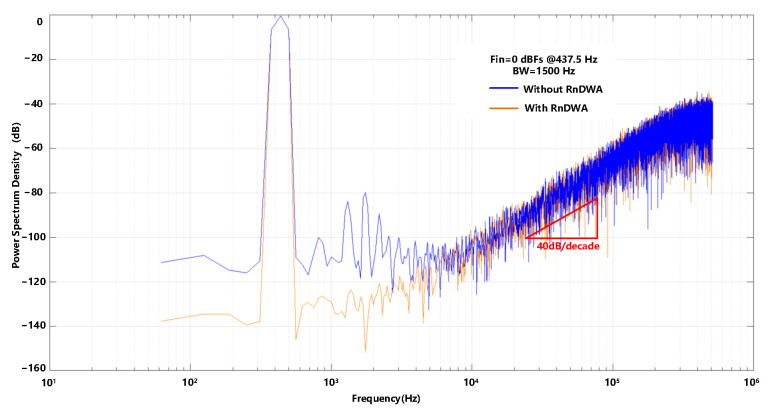
The power spectrum of the modulator with/without the RnDWA.

With the RnDWA, the mismatch error of DAC can be reshaped and pushed to high frequency. Taking into account the noise shaping, SNR in baseband for large OSR can be estimated by the following formula [25]:

(10) $$SNR=10log\{\frac{3OSR^{3}}{\pi^{2}\sigma_{u}^{2}}\frac{{\left(M+1\right)}^{2}}{M-1}\}$$

where *M* is the number of DAC units, OSR is the oversampling ratio, and $\sigma_{u}$ is the standard deviation of the random mismatch error of the units. With OSR = 341, M = 16, $\sigma_{u}=0.25\%$, a 135 dB in-band SNR can be obtained. Figure 14 shows the PSD of the modulator with and without the RnDWA.

### 3.4. Theoretical Analysis and Implementation of the Voltage Domain Module Technology

**Figure 15:**
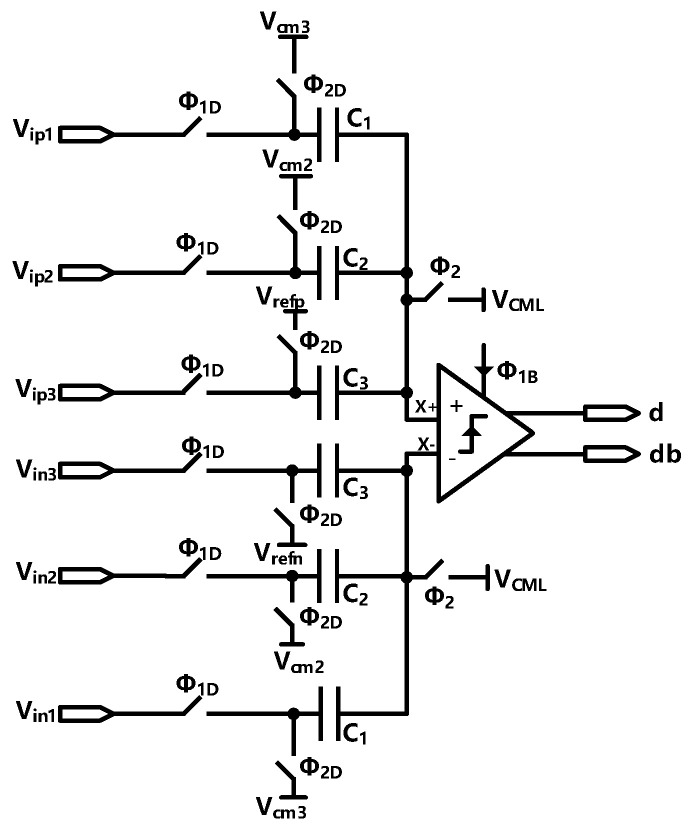
Schematic of the scaling voltage module.

The voltage domain technology has been used for saving power consumption of the quantizer and DEM logic circuits. The module makes use of the voltage scaling characteristics of the passive adder. The comparator is shown in Figure 15.

In phase $\phi_{2}$, the charge stored on the capacitors (take the positive of the comparator for example) can be expressed as:

(11) $$Q_{\phi_{2}}=\left(V_{refp}-V_{cm}\right)C_{1}+\left(V_{cm2}-V_{cm}\right)C_{2}+\left(V_{cm3}-V_{cm}\right)C_{3}$$

In the next phase $\phi_{1}$, the charge on the capacitor becomes

(12) $$Q_{\phi_{1}}=\left(V_{ip1}-V_{X_{+}}\right)C_{1}+\left(V_{ip2}-V_{X_{+}}\right)C_{2}+\left(V_{ip3}-V_{X_{+}}\right)C_{3}$$

Since the charge of the two phase is conserved, the equation can be written:

(13) $$\left(V_{refp}-V_{cm}\right)C_{1}+\left(V_{cm2}-V_{cm}\right)C_{2}+\left(V_{cm3}-V_{cm}\right)C_{3}=\left(V_{ip1}-V_{X_{+}}\right)C_{1}+\left(V_{ip2}-V_{X_{+}}\right)C_{2}+\left(V_{ip3}-V_{X_{+}}\right)C_{3}$$

We choose $C_{1}=\frac{3}{4}C,C_{2}=4C,C_{3}=C$, and we can derive that:

(14) $$V_{X_{+}}=\frac{3}{23}\left(V_{ip1}-V_{refp}\right)+\frac{16}{23}\left(V_{ip2}-V_{cm2}\right)+\frac{4}{23}\left(V_{ip3}-V_{cm3}\right)+V_{cm}$$

where $V_{cm}$ is the common mode voltage of the comparator, $V_{in1}$ is the input signal, $V_{refn}$ is the reference of the flash ADC, $V_{in2}$ is the output of the first integrator, $V_{in3}$ is the output of the second integrator, $V_{cm2}$ and $V_{cm3}$ are the common mode voltage of the output of the first and second integrators, respectively. Because the signal flowing in the loop only contains quantization noise, the output of the integrators is around 500 mV, so the max voltage of $V_{X+}$ is about 1.79 V. thus, the supply voltage of the comparator can be scaled to 1.8 V. and the different voltage of the comparator can be expressed as:

(15) $$\left(V_{X_{+}}-V_{X_{-}}\right)=\frac{3\left(V_{ip1}-V_{in1}\right)+16\left(V_{ip2}-V_{in2}\right)+4\left(V_{ip3}-V_{in3}\right)-4\left(V_{refp}-V_{refn}\right)}{23}$$

**Figure 16:**
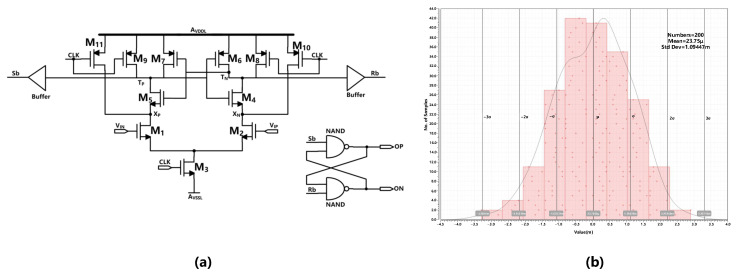
(**a**) Schematic of the comparator. (**b**) The Monte Carlo simulation result of the comparator’s offset.

To save power consumption, the dynamic comparator has been used. We performed a Monte Carlo simulation to evaluate the offset of the comparator. The schematic and simulation result is shown in Figure 16.

The simulation result shows that the mean offset voltage is 23.75 uV, and the σ (standard deviation of the offset) is 1.09 mV. As mentioned in [30], in order to control the error probability, the minimum resolution of the comparator should be greater than six times of the offset. The minimum resolution of the quantizer in this design is 112.5 mV which has a sufficient margin.

## 4. Results

**Figure 17:**
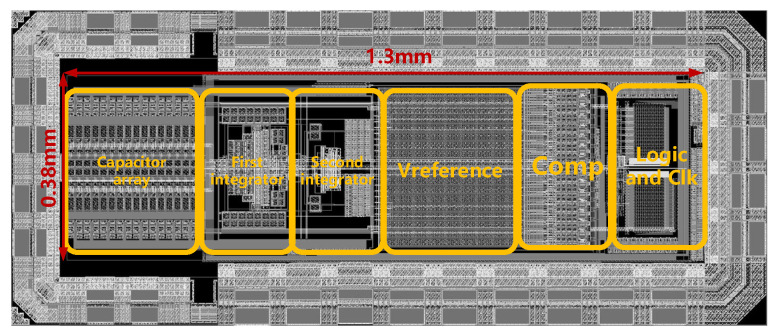
The layout circuit of the proposed modulator.

**Figure 18:**
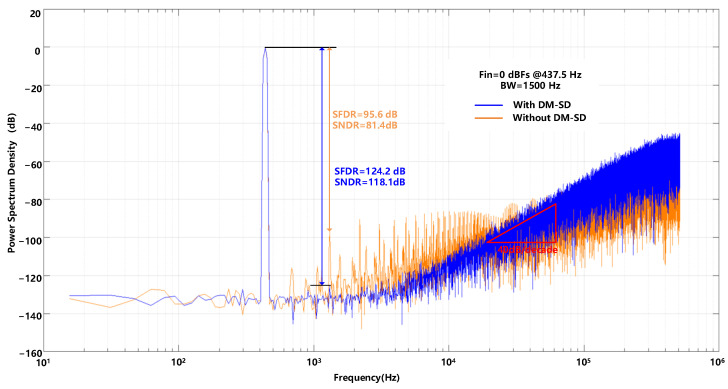
The power spectrum of the proposed modulator simulated with transient noise.

**Figure 19:**
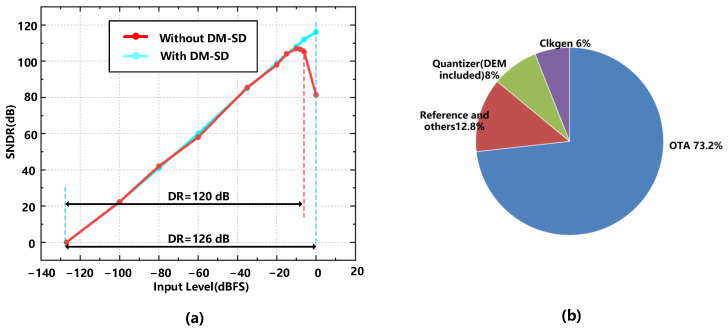
(**a**) SNDR versus input amplitude with/without DM-SD. (**b**) Total power consumption breakdown among circuit blocks.

The proposed sigma-delta modulator shown in Figure 17 is designed using 180 nm CMOS technology, occupying 0.49 mm${}^{2}$. Figure 18 shows the power spectrum with and without the DM-SD technology simulated with transient noise at a full-scale signal input (0 dBFS). The simulation results show that with a 0 dBFS, 437.5 Hz input signal, and 1500 Hz signal bandwidth, a 118.1 dB SNDR can be obtained. Figure 19a shows the SNDR versus the input amplitude with and without the DM-SD. The result shows that with the DM-SD technology, the DR can be extended to 126 dB. The power consumption diagram is shown in Figure 19b. Due to the use of voltage domain technology, the power consumption of the quantizer and the DEM circuit is reduced by about 30%. The total power consumption of the modulator is 1.6 mW. Table 2 compares the result of this work to state-of-the-art works. Among the listed reference, it is evident that our modulator achieves the highest SNDR (118.1 dB) and DR (126 dB). To evaluate the overall performance of the proposed ADC, Figure of Merit (FoMs) is used. It shows that our modulator achieves a competitive FoMs (177.8 dB) compared with other works.

## 5. Conclusions

In this paper, we propose a wide dynamic range and high-resolution discrete-time 2-order 4-bit sigma-delta modulator for non-invasive electroencephalogram acquisition. The modulator is designed with 180nm CMOS technology occupying a 0.49 mm${}^{2}$ area. A novel dynamic-modulated scaling-down technology has been proposed to extend the DR of the modulator. With the DM-SD technology, We achieve a 126 dB DR. We use the RnDWA and the low parasitic capacitance integrator technologies to optimize the performance of the modulator. With these techniques, we achieve a 118.1 dB SNDR. The total power consumption of the modulator is 1.6 mW, which benefits from the VDT technology, and a competitive FoMs (177.8 dB) is achieved.

## Figures and Tables

**Table 1:** The conditions of non-ideal circuit parameters to obtain required SNDR.

Non-ideality	Design Specifications	Unit
Finite Gain	≥60	dB
KT/C noise	≥4	pF
UGB	≥2	MHz
Slew rate	≥0.5	MV/s

**Table 2:** Performance summary and comparison with previous works.

Specification	This work	[31] IEICE Electron. Expr.	[15] IEEE Access	[32] IEICE Electron. Expr.	[23] Sci. China Inf. Sci.	[33] IEEE T CIRCUITS-II
Year	2022	2018	2021	2019	2022	2021
Architecture	DT ΣΔ	DT ΔΣ	RB DT ΣΔ	DT ΣΔ	DT ΣΔ	DT ΣΔ
Tech (μm)	0.18	0.35	0.18	0.04	0.18	0.11
Supply (V)	5&1.8	5	1.8	0.9	5	1.5
Power (μW)	1600	12,600	2200	790	2750	62.43
BW (Hz)	1500	1200	25,000	100,000	125	2000
SNDR (dB)	118.1	105.2	106.1	87.3	110	93.9
DR (dB)	126	113.7	-	88.1	124	96.3
$F_{S}$ (MHz)	1.024	-	12.8	25.6	1.024	60
$F_{O}M_{S}$ (dB) ^1^	**177.8**	163.4	176.5	168	170.6	172

^1^$F_{O}M_{S}=\mathrm{SNDR}+10\log_{10}(\frac{BW}{\mathrm{Power}})$ [15].

## Abbreviations

The following abbreviations are used in this manuscript:

ADC	Analog to Digital Converter
AFE	Analog Front End
BCI	Brain Computer Interface
CT	Continue Time
CMFB	Common Mode Feedback
CMOS	Complementary Metal-Oxide-Semiconductor Transistor
DAC	Digital to Analog Converter
DEM	Dynamic Element Match
RnDWA	Randomized Data Weighted Averaging
DM-SD	Dynamic-Modulated Scaling-Down
DR	Dynamic Range
DT	Discrete Time
EEG	electroencephalogram
ENOB	Effective Number of Bits
FFMC	Feed-Forward Miller Compensation
OSR	Over-Sampling Ratio
OTA	Operational Transconductance Amplifier
PVT	Power, Voltage, and Temperature
SNR	Signal-to-Noise Ratio
SNDR	Signal-to-Noise plus Distortion Ratio
SAR	Successive Approximation Register
STF	Signal Transfer Function
SR	Slew Rate
THD	Total Harmonic Distortion
UGB	Unity Gain bandwidth
VDT	Voltage Domain Technology
